# Physical activity dimensions after stroke: patterns and relation with lower limb motor function

**DOI:** 10.1186/s12984-021-00960-x

**Published:** 2021-12-11

**Authors:** Hanneke E. M. Braakhuis, Monique A. M. Berger, Ruben G. R. H. Regterschot, Erwin E. H. van Wegen, Ruud W. Selles, Gerard M. Ribbers, Johannes B. J. Bussmann, the PROFITS research group, Carel Meskers, Gert Kwakkel, Erwin E. H. van Wegen, Rinske Nijland, Aukje Andinga, Valentijn Zonjee, Muriel Koolstra-Rutgers, Renske van den Berg-Vos

**Affiliations:** 1 Department of Rehabilitation Medicine and Physical Therapy, Erasmus MC University Medical Center, PO Box 2040, 3000 CA Rotterdam, The Netherlands; 2 Department of Plastic and Reconstructive Surgery, Erasmus MC University Medical Center, Rotterdam, The Netherlands; 3 Rijndam Rehabilitation, Rotterdam, The Netherlands; 4 Faculty of Health, Nutrition and Sport, The Hague University of Applied Sciences, The Hague, The Netherlands; 5 Department of Biomedical Signals and Systems, University of Twente, Enschede, The Netherlands; 6 Department of Rehabilitation Medicine, Amsterdam Neuroscience and Amsterdam Movement Sciences, Amsterdam UMC, Vrije Universiteit Amsterdam, Amsterdam, The Netherlands

**Keywords:** Stroke, Motor function, Physical activity, Accelerometry

## Abstract

**Background:**

Stroke survivors show deteriorated physical functioning and physical activity levels. Physical activity levels of stroke survivors are generally low. It is increasingly recognized that physical activity is a multidimensional construct that cannot be captured in a single outcome. In-depth insight into multidimensional physical activity patterns may guide the development and timing of targeted rehabilitation interventions. This longitudinal cohort study explored how multidimensional physical activity outcomes develop during recovery in the subacute phase after stroke and if changes in physical activity were correlated to recovery of lower limb motor function.

**Methods:**

Patients were recruited during inpatient rehabilitation. At 3, 12, and 26 weeks post-onset, motor function was measured by the Fugl-Meyer Lower Extremity Assessment (FMA-LE). Physical activity was measured with the Activ8 accelerometer in multiple outcomes: counts per minute during walking (CPM_walking_; a measure of Intensity), number of active bouts (Frequency), mean length of active bouts (Distribution) and % of waking time in upright positions (Duration). Generalized estimating equations (GEE) were used to study changes in physical activity over time and the relation with the change in lower limb motor recovery.

**Results:**

Thirty-nine patients (age 56 ± 9, 77% male, 89% ischemic stroke) were included. GEE models showed a significant main effect of time for PA Intensity (+ 13%, p = 0.007) and Duration (+ 64%, p = 0.012) between 3 and 12 weeks. Motor function did not show a significant effect in all PA models across the 3 timepoints (p > 0.020). A significant interaction effect of time × motor function was observed (p < 0.001).

**Conclusions:**

Patterns of PA recovery depend on the PA dimensions: PA Intensity and Duration increased mostly between 3 and 12 weeks post-stroke, whereas Frequency and Distribution did not show substantial changes. Further, no strong associations with motor recovery and high inter-individual variability were documented, which underlies the need to consider factors specific to the disease, the individual patient and the context.

## Introduction

Approximately two-thirds of stroke survivors experience physical functioning problems, resulting in low levels of participation in physical activity (PA) [1, 2]. Pursuing a physically active lifestyle is important because it reduces the risk for recurrent strokes, and it is linked to better functional capacity, quality of life, and overall life satisfaction [3, 4]. Therefore, from very early on post-stroke, one of the rehabilitation targets is optimizing patients' levels of PA [5–7].

In the last decade, objective measurement of PA is increasingly used in stroke studies, with accelerometry as the dominant technology [8]. Although accelerometry is relatively simple in itself, the interpretation and comparison of data are complex due to variable methods and devices. In addition, multiple outcome measures are reported, affecting the conclusions [8–10]. For example, Sanchez et al. [11] reported the mean duration of walking bouts after stroke and showed that it did not differ from healthy controls. In contrast, other studies showed that the average walking time and the daily number of steps were significantly lower in stroke survivors [11–13]. It is increasingly recognized that PA is multidimensional, including dimensions such as Intensity, Frequency, Duration and Distribution [8, 10, 14]. Therefore, clinically relevant information on PA cannot be captured in one outcome and reporting multiple outcomes concurrently preferred [8, 10, 15].

Longitudinal studies describing changes in multiple dimensions of PA post-stroke are scarce. Two longitudinal studies found different patterns of multiple dimensions, for example; frequency and time in short, long, low and moderate intensity bouts [16, 17]. Both studies started their measurements after discharge from rehabilitation, between 3 weeks and 4 months post-stroke. However, especially in the subacute phase (between 7 days and 6 months), measurements at fixed time points post-stroke are recommended due to the timing of several biological recovery processes [18]. Insight into the multidimensional PA patterns within the subacute phase is needed since it may guide appropriate timing and development of targeted interventions in rehabilitation [19–21].

PA patterns after stroke may be influenced by the level of motor recovery of a patient, since the performance of daily activities, such as walking, requires sufficient motor function, which is dependent on synergies [22]. However, a cross-sectional study showed no association between motor function and self-reported PA [23]. To date, it is unknown what the longitudinal relation is between motor function and PA measured in multiple dimensions with accelerometry. This longitudinal cohort study explored how multidimensional physical activity outcomes develop during recovery in the early and late subacute phase after stroke and how this related to changes in motor recovery.

## Methods

### Study design and participants

This is a longitudinal observational cohort study. Patients were included < 3 weeks post stroke in this sub-study from Rijndam Rehabilitation (Rotterdam, The Netherlands) if they suffered from an ischemic or hemorrhagic stroke with a paretic arm or leg (defined as NIHSS 5A/B or 6A/B 4 ≥ score > 0). Other inclusion criteria were (i) 18 years or older, (ii) a Mini Mental State Examination (MMSE) score > 19, and (iii) ability to sit at least 30 min with back support. Patients were screened by a trained research assistant between September 2016 and June 2019. All patients included in this study received the usual inpatient rehabilitation care program at Rijndam Rehabilitation. All patients gave their written informed consent, and the study was approved by the Medical Ethics Committee of Erasmus MC University Medical Center Rotterdam, The Netherlands (MEC-2015-687).

### Procedures

Measurements were conducted at three fixed time points post-stroke; 3 (T1), 12 (T2) and 26 weeks (T3) [24]. Demographic and clinical characteristics were collected at the time of inclusion. At each time point, a trained assessor conducted all tests. During the first measurement (T1), patients were visited during inpatient rehabilitation; The measurements at 12 and 26 weeks took place during either inpatient or outpatient rehabilitation or at home. If a patient was discharged from inpatient services, the patient was visited at home.

### Measures

#### Motor function

Motor function was determined by the Fugl Meyer Lower Extremity Assessment (FMA-LE) administered at 3, 12, and 26 weeks post-stroke [25]. The FMA-LE assesses motor function of the lower extremity based on diverse tasks, concerning reflex activity, movement within and outside synergy patterns, speed and coordination. The FMA-LE consists of 17 items, with a maximum score of 34 points. Each item was scored on a 3-point scale (0 = cannot perform, 1 = can partially perform, 2 = can fully perform). A higher score represents a higher level of motor function.

#### Physical activity

PA was measured by the Activ8, which is a small (30*32*10 mm) and light-weight (20 g) triaxial accelerometer that can validly and continuously measure daily PA of individuals after stroke [26]. The Activ8 was attached to the front of the thigh of the non-affected leg of the patient with TegadermTM skin tape. This waterproof attachment allowed patients to swim and shower while wearing the device. The patients wore the Activ8 for 7 consecutive days. In addition to the PA monitoring, the participants were asked to report waking hours each day in a logbook to check whether this corresponded with the registration by the Activ8. PA assessments were considered valid if data from at least 10 h of waking hours per day were available over 5 days [27].

The output of the Activ8 monitor consists of time spent in six categories of body postures and movements (lying, sitting, standing, walking, running and cycling) within an epoch length of 30 s [14]. In each epoch, the number of movement counts is calculated for each category, representing the amount of movement within that epoch. The movement intensity van be calculated for each category, by dividing the number of movement counts by the time spent in a category. Standing, walking, running and cycling were merged into upright activities, while the same activities minus standing were classified as active activities. If a 30-s epoch consisted of > 80% of active activities, such an epoch was classified as active. If a time period of at least 4 subsequent active epochs occurred (i.e. a 2-min period at least), such a period was classified as an active bout.

Matlab R2014b was used to process the time and counts of the postures and movements into different outcomes representing four distinct dimensions of PA:

- *Intensity* counts per minute during walking (CPM_walking_) [28]. Walking is the most common and important movement for stroke survivors in daily activities and participation in society [29–31].
- *Frequency* the number of active bouts (N Bout_active_)
- *Distribution* the mean length of active bouts (ML Bout_active_) represented the distribution of PA and was calculated by the sum of the length of all active bouts divided by the number of active bouts.
- *Duration* the relative time (% Upright) in upright postures and movements represented duration of PA and was calculated by the sum of the duration in upright movements, divided by the total waking time multiplied by 100%.

All outcome measures were averaged per day by dividing by the number of days that contained valid measurements.

### Statistical analyses

Statistical analyses were performed in RStudio (version 1.2.50001, RStudio, Inc.). Baseline characteristics, motor function and PA outcomes were described by means and standard deviations with minimal and maximal values for continuous variables and frequencies and percentages for categorical variables.

Marginal modelling with Generalized Estimating Equations (GEE) was used to detect longitudinal changes since it controls for correlations between repeated measurements [32]. All four PA outcomes were used as dependent variables in the GEE models. Time was set as an independent factor with three levels (3, 12, 26 weeks). For all models, an identity link function was used according to the distribution of the PA outcomes. The choice of the most suitable working correlation matrix was based on the lowest quasi-likelihood under the independence model criteria (QIC) [32].

First, to detect changes in multidimensional PA over time, a univariate GEE model with only time as a predictor was developed for each PA outcome. After that, to investigate the relation with motor recovery, other multivariate GEE models, including time, motor function and an interaction between time and motor function were developed. The interaction term assessed the association between PA outcomes and changes in motor function over time. These GEE models were conducted with stepwise approach; first, a full model was developed with time, motor function and the interaction between time and motor function. Second, if the interaction term showed no added value, it was deleted from the model.

Since PA is measured in four domains, we used Bonferroni for correcting for multiple testing, considering p < 0.0125 as significant. If a significant main effect of time was observed, post-hoc comparisons with a Bonferroni correction was conducted. Post-hoc analyses were considered significant at p < 0.05.

## Results

### Participants

**Fig. 1:**
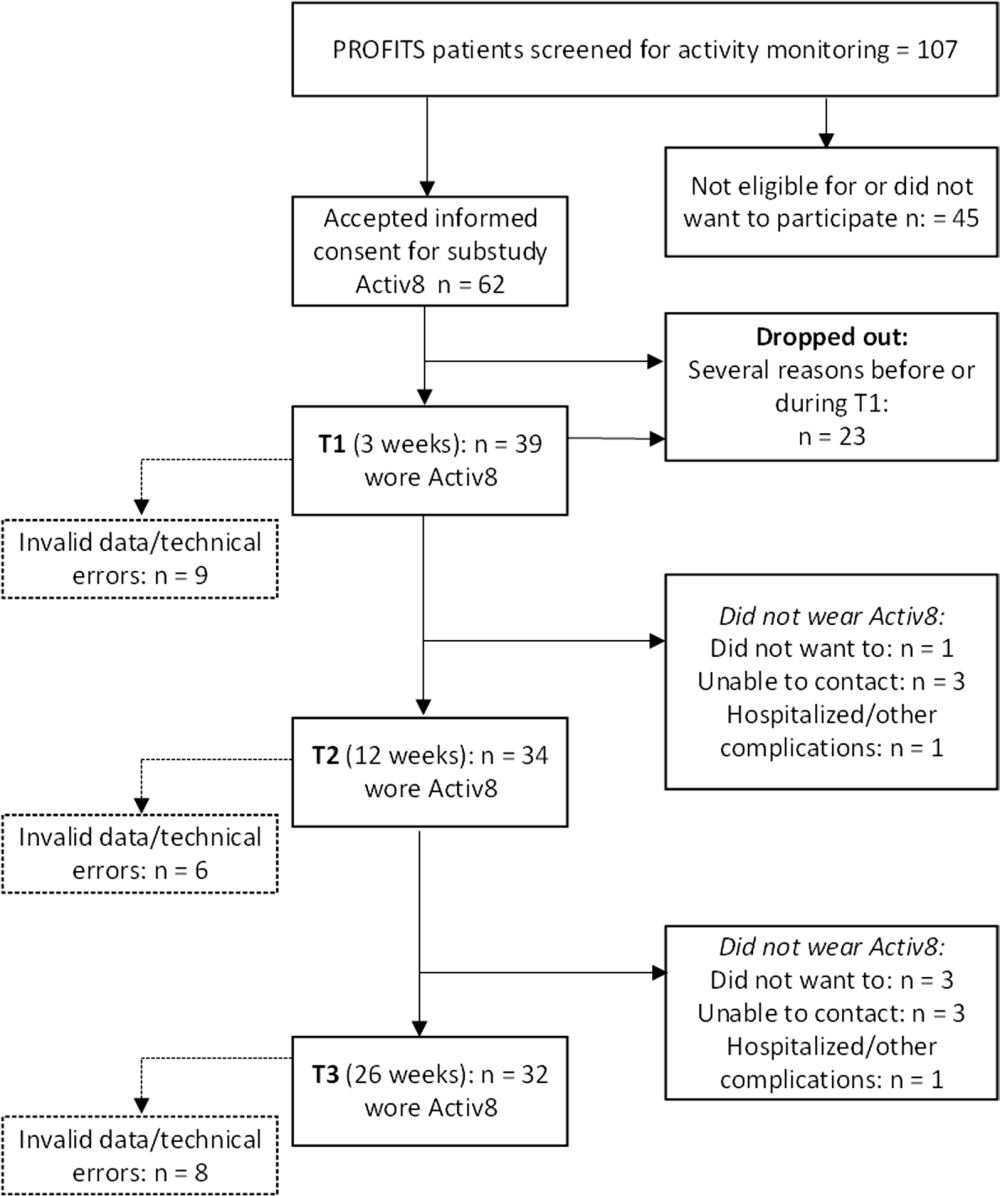
Flowchart of inclusion of patients

Figure 1 shows the flow of inclusion of patients. Sixty-two patients accepted informed consent. Twenty threepatients withdrew before or during the first measurement (T1) and were excluded. Reasons for withdrawing were amongst others; withrew from study due personal reasons, wrong diagnosis, hospitalization, and early discharge. Thirty-nine patients were included in further analyses. The number of valid measurements that were included in the analysis was n = 30 at T1, n = 28 at T2 and n = 24 at T3. Baseline characteristics of the patients within the study sample at baseline (n = 39) are shown in Table 1.

**Table 1:** Baseline characteristics of patients included in Activ8 measurements (N = 39)

Age (years, mean ± SD, min–max)	56 ± 9 (37–75)
Sex (male n, %)	30, 77%
Type of stroke (hemorrhagic/ischemic, n, %ischemic)	4/35, 90%
Time between stroke and admission to inpatient rehabilitation (days mean ± SD, min–max)	11 ± 6 (0–22)
Length of inpatient rehabilitation (days, mean ± SD, min–max)	59 ± 34 (9–120)
Barthel Index (mean ± SD, min–max)	15 ± 4 (7–20)
Motricity Index Lower Extremity (mean ± SD, min–max)	64 ± 29 (0–100)
Berg Balance Scale (mean ± SD, min–max)	36 ± 16 (4–56)
Fugl Meyer lower extremity (mean ± SD, min–max)	22 ± 10 (4–33)

### Longitudinal changes of physical activity

**Fig. 2:**
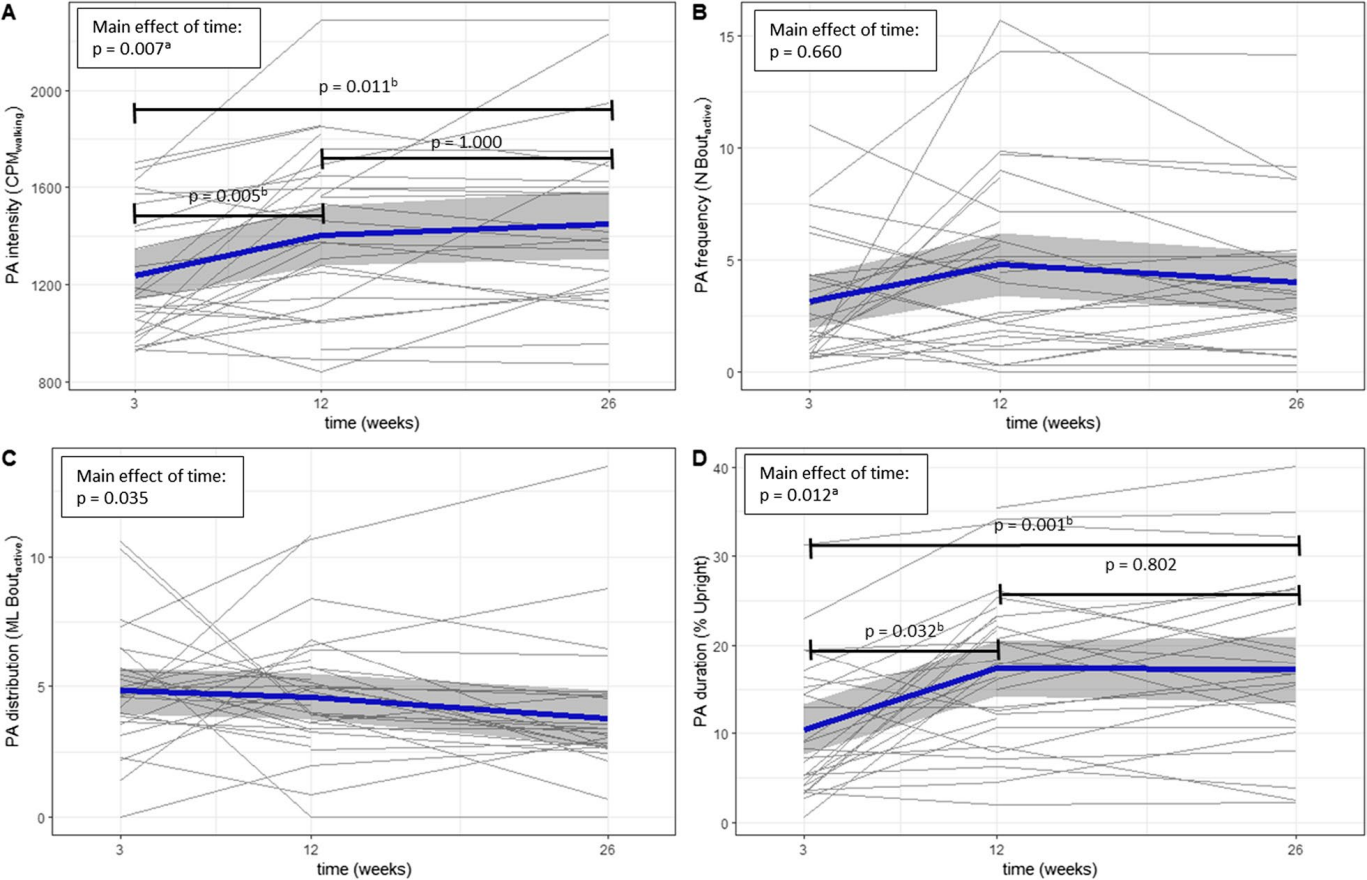
Individual and mean changes of physical activity (PA) intensity, frequency, distribution and duration from 3 to 26 weeks post-stroke with p-values of of post-hoc analyses between time points from the univariate generalized estimating equations (GEE) models. NOTE: Grey lines represent PA of individuals, blue lines represent mean PA and grey band represent 95% CI, ^a^p < 0.0125 for main effect, ^b^p < 0.050 for post-hoc analyses between time points

The mean waking time used for Activ8 measurements was 14h19min ± 1h6min per day. Figure 2 shows PA changes per individual and mean change of the sample. Additionally, Fig. 2 shows the results of the univariate GEE models with only time as a predictor. After Bonferroni correction, a main effect of time was observed for PA intensity (p = 0.007) and PA duration (p = 0.001) but not for PA frequency (p = 0.660) and distribution (p = 0.035). Post-hoc analyses showed a significant increase (+ 13%) of PA intensity between 3 and 12 weeks (p = 0.005) and significant increase (+ 64%) of PA duration between 3 and 12 weeks (p = 0.032).

### Longitudinal relation between physical activity and motor function

Average FMA-LE at 3 weeks was 22 ± 10, at 12 weeks 27 ± 7 and at 26 weeks 27 ± 6. Table 2 shows the results of the four multivariate GEE models, including time, motor function and time × motor function. In the multivariate GEE analyses, main effects of time were observed in the PA intensity (p = 0.007) and duration (p = 0.001) model (Table 2). Post-hoc analyses between time points showed a significant effect for duration between 3 vs. 26 weeks (p < 0.021). No effect (p < 0.013) for motor function was observed in all PA models (PA Intensity; p = 0.032, Frequency; p = 0.020, Distribution; p = 0.021, Duration; p = 0.121).

**Table 2:** Results of multidimensional physical activity (PA) in the multivariate generalized estimating equations (GEE) models with post hoc analyses between 3 vs. 12. 3 vs. 26 and 12 vs. 26 weeks post-stroke

	β	SE	p-value	p-value of the main effect of time	Post-hoc between time points, p-value		
					3 vs. 12 weeks	3 vs. 26 weeks	12 vs. 26 weeks
PA intensity (CPM_walking_*10^3^)							
Time (12 weeks)	176	853	0.040	0.007^a^	0.118	0.254	1.000
Time (26 weeks)	153	887	0.085				
FMA-LE	10.7	498	0.032				
PA Frequency (N Bout_active_)							
Time (12 weeks)	− 0.51	1.55	0.744	0.032	–	–	–
Time (26 weeks)	− 1.21	1.21	0.318				
FMA-LE	0.17	0.07	0.020				
PA distribution (ML Bout_active_)							
Time (12 weeks)	− 5.88	1.78	0.001^a^	0.035	–	–	–
Time (26 weeks)	− 7.12	1.80	< 0.001^a^				
FMA-LE	− 0.11	0.05	0.021				
Time (12 weeks) × FMA-LE	0.22	0.07	0.001^a^				
Time (26 weeks) × FMA-LE	0.23	0.07	0.001^a^				
PA duration (% Upright)							
Time (12 weeks)	4.83	2.47	0.051	0.001^a^	0.153	0.021^b^	1.000
Time (26 weeks)	5.91	2.20	0.007^a^				
FMA-LE	0.24	0.15	0.121				

*PA* physical activity, *FMA-LE* Fugl Meyer Assessment Lower Extremity as a measure of motor function, *n/a* not applicable

^a^p < 0.013 for main effect

^b^p < 0.050 for post-hoc analyses between time points

## Discussion

This study showed that PA Intensity and Duration improved between three and twelve weeks post stroke whereas PA Frequency and Distribution did not show significant change during the subacute phase after stroke. Overall, the relation with motor recovery was absent or weak. In all PA dimensions, high inter-individual variability, both cross-sectional and over time was observed.

It is generally known that the most considerable improvement in post-stroke physical functioning is by spontaneous recovery occurring most strongly within the first 5 to 6 weeks, and by intensive rehabilitation therapy within the first 3 months post stroke [5, 19]. Our study showed significant improvements in PA Intensity and PA Duration from 3 to 12 weeks post-stroke, with a plateau thereafter. In other words, patients increased spending time upright and walked more intensively. PA Intensity, measured by accelerometercounts during walking, indicates walking speed, and has been shown a sensitive measure for detecting clinically important changes [28, 33]. In contrast, no increase was observed in the bout-specific outcomes of PA Frequency (bout number) or PA Distribution (bout length), suggesting that the passage of time after stroke did not lead to more persistent and prolonged physical activities of two minutes or more. Therefore, it seems that the evaluation of temporal PA changes is sensitive to the selected outcome measure, which is in line with the results of Mahendran et al. [16]. Since until now, no consensus on the best post-stroke PA measures has been recommended [8], we recommend measuring and reporting multiple dimensions of PA. Besides giving a complete overview of patients' PA, it will also contribute to a better understanding and a well-grounded selection of future outcomes that are sensitive to change post stroke.

Only the PA Distribution model showed significant interaction effects between motor function and time at 12 weeks and 26 weeks after stroke, meaning that patients with increasing motor function seem to be more persistent in uninterrupted activity as time progresses. However in general, no or at best weak associatons between PA en motor function were found in the multivariate GEE models. One explanation is that overall, the FMA-LE scores in our study sample were relatively high, and the changes over time relatively small. At three weeks, mean FMA-LE was 22 ± 10. According to Kwong et al. [34], a score of 21 or higher represents a high level of motor function in stroke survivors. It is possible that for these patients, substantial spontaneous recovery occurred before the first measurement and no large nor clinically relevant FMA-LE changes were taken into account in our longitudinal analysis. FMA-LE may not be sensitive enough to detect small increments in motor function in patients with a relatively high level of motor function. Also, learned compensatory strategies to overcome motor impairments might have distorted the relationship [35]. The weak relationship between physical activity and motor recovery supports the importance of collecting objective information on a patients’ performance in their own context, such as accelerometer-based PA, is relevant in addition to other clinical tests.

Another remarkable finding was large within and between-subject variability that was observed in our study (Fig. 2). To illustrate, the relative time spent in upright positions at 26 weeks after stroke ranged from 1 to 40% of the day, representing eight minutes a day to more than five hours. Comparable ranges were found in PA intensity, frequency and distribution. This variability might be the result of the varying demographics, functional level (Table 1), and other factors such as cognitive impairments, and pre-stroke lifestyle, physical and social environment [23]. The high intra-individual variability underscores the urge for an individual approach in rehabilitation research and practice [5, 36].

Unique in our study was the measurements at fixed time points post onset aligned with the underlying recovery mechanisms of body structures and functions [24]. In contrast, other longitudinal PA studies [16, 17] measured at time points relative to time of admission to or discharge from rehabilitation, reflecting a process of care [18]. Future research should reveal if measurements of PA changes based on both approaches differ and what is most informative for appropriate timing of interventions. To date, optimal timing of interventions after stroke is still a challenge [18, 21].

Although similar to earlier studies on post-stroke PA [16, 37, 38], a limitation of this study was the relatively small sample size. Therefore, the results of the regression models should be interpreted with caution. Another limitation was the amount of missing data resulting from device failures and subject compliance. Future developments—e.g., smaller sensors, body posture and movement detection from wrist-worn devices—might improve compliance in future studies. Nevertheless, GEE analyses appropriately handles at-random missing data. Also, the choice of outcome measures may have influenced our results. To the best of our knowledge, we chose four theoretically different physical activity measures that are still easy to interpret from many available possibilities.

## Conclusion

Our study showed that patterns of PA recovery depend on the PA dimensions: PA Intensity and Duration increased mostly between three and twelve weeks post-stroke, whereas Frequency and Distribution did not show substantial changes. Further, we observed high inter-individual variability and no, or at best weak associations between PA dimensions and motor recovery. The observed differences in PA patterns underline the importance of capturing multiple PA dimensions and considering factors specific to the disease, the individual patient and the context.

## Data Availability

The datasets generated and/or analysed during the current study are currently not publicly available due other PROFITS studies that are still in progress.
